# Epigenetic Effects of Prenatal Stress on 11β-Hydroxysteroid Dehydrogenase-2 in the Placenta and Fetal Brain

**DOI:** 10.1371/journal.pone.0039791

**Published:** 2012-06-26

**Authors:** Catherine Jensen Peña, Catherine Monk, Frances A. Champagne

**Affiliations:** 1 Department of Psychology, Columbia University, New York, New York, United States of America; 2 Departments of Psychiatry and Obstetrics & Gynecology, Columbia University, New York, New York, United States of America; Fudan University, China

## Abstract

Maternal exposure to stress during pregnancy is associated with significant alterations in offspring neurodevelopment and elevated maternal glucocorticoids likely play a central role in mediating these effects. Placental 11β-hydroxysteroid dehydrogenase type 2 (*HSD11B2*) buffers the impact of maternal glucocorticoid exposure by converting cortisol/corticosterone into inactive metabolites. However, previous studies indicate that maternal adversity during the prenatal period can lead to a down-regulation of this enzyme. In the current study, we examined the impact of prenatal stress (chronic restraint stress during gestational days 14–20) in Long Evans rats on *HSD11B2* mRNA in the placenta and fetal brain (E20) and assessed the role of epigenetic mechanisms in these stress-induced effects. In the placenta, prenatal stress was associated with a significant decrease in *HSD11B2* mRNA, increased mRNA levels of the DNA methyltransferase *DNMT3a*, and increased DNA methylation at specific CpG sites within the *HSD11B2* gene promoter. Within the fetal hypothalamus, though we find no stress-induced effects on *HSD11B2* mRNA levels, prenatal stress induced decreased CpG methylation within the *HSD11B2* promoter and increased methylation at sites within exon 1. Within the fetal cortex, *HSD11B2* mRNA and DNA methylation levels were not altered by prenatal stress, though we did find stress-induced elevations in *DNMT1* mRNA in this brain region. Within individuals, we identified CpG sites within the *HSD11B2* gene promoter and exon 1 at which DNA methylation levels were highly correlated between the placenta and fetal cortex. Overall, our findings implicate DNA methylation as a mechanism by which prenatal stress alters *HSD11B2* gene expression. These findings highlight the tissue specificity of epigenetic effects, but also raise the intriguing possibility of using the epigenetic status of placenta to predict corresponding changes in the brain.

## Introduction

In humans, the experience of stress during pregnancy is associated with increased risk of preterm birth, reduced birth weight, and smaller head circumference [1]–[3] and has been implicated in the heightened risk of metabolic and psychiatric disorders [4]–[7]. The long-term consequences of maternal stress during gestation for offspring neurobiological and physiological functioning have likewise been demonstrated in laboratory studies using rodents [8]–[11], suggesting a causal link between this form of prenatal adversity and various developmental outcomes. Though there may be multiple pathways through which maternal stress during pregnancy shapes offspring development, stress-induced elevations in maternal glucocorticoids have emerged as a primary mechanism of prenatal stress effects [12], supported by studies in rats in which the effects of prenatal stress are prevented amongst adrenalectomized females [13].

In mammals, the placenta serves as a critical interface between maternal and fetal physiology and forms a barrier to maternal glucocorticoids [14], [15]. Human self-reports of stress during pregnancy are associated with increased circulating cortisol, but only 10–20% of maternal cortisol passes to the fetus [16], [17]. This barrier function of the placenta is achieved through the actions of the enzyme 11β-hydroxysteroid dehydrogenase type 2 (11ß–HSD2), which converts the glucocorticoids cortisol/corticosterone into the inactive metabolites cortisone/11β-dehydrocorticosterone, thus preventing the activation of glucocorticoid receptors [14]. In contrast, the enzyme 11ß–HSD1 converts inactive glucocorticoids to cortisol/corticosterone [18], [19]. Targeted gene deletion and pharmacological studies suggest a functional consequence of 11ß–HSD for the development of the hypothalamic-pituitary adrenal (HPA) response to stress. In mice, mutation of the *HSD11B2* gene leads to hypertension, excess mineralocorticoid activity, and increased anxiety-like behavior in adulthood [20] whereas *HSD11B1* mutation leads to attenuated negative-feedback of the HPA response to stress and improved cognitive performance in aging [21]. Pharmacological inhibition of 11ß–HSD2 during pregnancy or administration of dexamethasone, a synthetic glucocorticoid that is not metabolized by 11ß–HSD2, leads to molecular and neurobiological changes within the HPA axis associated with increased stress responsivity and anxiety-like behavior in adulthood [22], [23]. These glucocorticoid programming effects may also be evident in humans, consequent to prenatal betamethasone exposure or inhibition of 11ß–HSD2 through elevated glycyrrhizin consumption during pregnancy [24], [25]. These studies suggest that the regulation of placental 11ß–HSD2 levels may be a mechanistic link between the experience of maternal gestational stress and long-term health outcomes in offspring.

There is increasing evidence that maternal adversity during pregnancy may lead to a down-regulation of 11ß–HSD2. In rats, chronic restraint stress during gestational days 11–20 (equivalent to the 2^nd^–3^rd^ trimester of human pregnancy) was found to decrease placental 11ß–HSD2 enzymatic activity and decrease mRNA levels of this gene [26]. Similarly, rat dams that are food restricted from gestational days 10–20, a manipulation that increases maternal plasma corticosterone and induces similar phenotypes to those observed following prenatal stress, have reduced placental protein levels of 11ß–HSD2 [27], [28]. In humans, heightened maternal anxiety (assessed just prior to parturition) was found to be negatively correlated with placental *HSD11B2* mRNA levels [29]. Reduced placental *HSD11B2* mRNA levels have also been found associated with intrauterine growth retardation and pre-term birth [30], suggesting that the transcriptional activity of this enzyme may be predicted by maternal adversity and predictive of high risk birth outcomes.

Regulation of gene expression through epigenetic mechanisms – factors that alter gene transcription without altering DNA sequence - is being increasingly explored within the context of environmentally-induced changes in neurobiology, metabolism, and disease risk [31]–[33]. Epigenetic changes involving DNA methylation (at cytosine-guanine sequences – CpG sites), post-translational modification of histone proteins, and microRNAs can have dynamic effects on gene expression and may also account for the stability of experience-dependent effects on transcription [34]–[38]. Epigenetic dysregulation has been associated with prenatal intrauterine growth restriction [39] and disease in both humans and rodents [39]–[41]. Moreover, there is increasing evidence that maternal adversity during gestation can induce epigenetic changes in placenta and fetal tissues that may account for the heightened HPA reactivity amongst offspring [9].

In the current study, we examined the impact of gestational maternal stress in pregnant rats on the transcription and DNA methylation of the *HSD11B2* gene to determine whether epigenetic factors may account for the down-regulation of placental *HSD11B2* function in response to stress. An important consideration in studies of environmental-induced epigenetic effects for which there has been limited empirical investigation, is tissue specificity, and here we compared the impact of gestational maternal stress on CpG methylation in placenta as well as fetal hypothalamus and cortex. Moreover, we explored the potential role of the DNA methyltransferases *DNMT1* and *DNMT3a* – enzymes that promote DNA methylation [42], [43] – in these tissues, to determine the possible mechanistic pathways through which stress-induced epigenetic variation is achieved. Finally, we also assessed the feasibility of using placental epigenetic variation in the *HSD11B2* gene to predict DNA methylation levels of this gene in the fetal hypothalamus and cortex. This study provides new insight into the molecular basis of the effects of maternal adversity and highlights issues that are critical for the study of epigenetic effects and the translation of epigenetic analysis to studies of human prenatal exposures.

## Methods

### Animals & Husbandry

16 female and 8 male Long Evans rats (purchased from Charles River) were maintained on a 12:12 hour light-dark schedule with white lights on at 0800 h and off at 2000 h and housed 2 per cage (same-sex) in 26×50×22 cm polycarbonate cages in the animal facility at the Department of Psychology, Columbia University. Food and water were available *ad libitum* and replenished daily by animal care staff at 0900. Animals were habituated to the facility for 2 weeks prior to mating. Pair-housed virgin females were mated for one week. Timing of the pregnancy was confirmed by the presence of a vaginal plug (designated as gestational day 0). All procedures were performed in accordance with guidelines of the NIH regarding the Guide for the Care and Use of Laboratory Animals and with the approval of the Institutional Animal Care and Use Committee (IACUC) at Columbia University.

### Gestational Stress

Within the sample of 16 mated females, 12 females became pregnant following the 1-week mating period. At gestational day 14, all pregnant females were singly-housed and assigned to either non-stress (control, n = 6) or stress (n = 6) treatment conditions. Control females were left undisturbed throughout gestational days 14–20. Stress females were exposed to restraint stress, through placement in a 19×29×12 cm cage (which prevented vertical and horizontal movement) for 1-hour/day from gestational days 14–20. The timing of stress exposure was randomized to prevent habituation. At gestational day 20, pregnant dams were sacrificed 1 hour after restraint stress through rapid decapitation and trunk blood was collected for assay of corticosterone levels. Plasma corticosterone was assayed using an RIA kit (MP Biomedicals) and this assay confirmed elevated levels of corticosterone in stressed compared to control dams [control: 381.67 ng/ml±33.41; stress: 623.33 ng/ml±36.02; t(10) = 4.92, p<.001].

### Tissue Dissection

Feti and placenta were extracted *via* caesarean section at gestational Day 20. Pups were decapitated and whole brains extracted. Placenta and fetal brains were snap-frozen, and stored at −80C until further processing. Cortex and hypothalamus of E20 fetal brains were dissected in a cryostat cooled to −20C. Placenta samples were dissected such that a pie slice including both the basal zone and inner labyrinth zone was used. Samples were weighted and homogenized in 700 µl lysis buffer RLT-Plus (Qiagen) with 0.1% β-mercaptoethanol using a tissue homogenizer (Omni) for 15–20 seconds. To analyze both mRNA and DNA methylation from the same sample, RNA and DNA from fetal cortex, hypothalamus, and placenta samples were isolated simultaneously using a dual RNA/DNA extraction kit (Qiagen). cDNA was synthesized using a reverse transcription kit (Applied Biosystems) according to manufacturer’s protocol. Samples were stored at −20C until further processing. Subsequent analysis of gene expression and CpG methylation was conducted using tissue from 1–2 offspring each of 4 control and 4 stress dams. Selection was based on average pup weight at the time of sacrifice. Only litters in which average pup weight was greater than 2.5 g were included in this sample and average pup weight did not differ significantly between treatment groups.

### Gene Expression Analysis

Tissue (hypothalamus, cortex, placenta) from 2 pups per litter from 4 control and 4 stress dams were included in the gene expression analyses. Relative gene expression was measured by real-time quantitative PCR on a 24-well 7500Fast qPCR thermocycler using SybrFast (Applied Biosystems) with standard amplification and Ct calculation protocols by Applied Biosystems. All primers were designed to span exons and were tested for specificity (single melt curve peak) and efficiency (87–105%). Primer pairs are included in **Table 1**. Calculations of relative gene expression of 11β-hydroxysteroid dehydrogenase type 2 (*HSD11B2*), 11β-hydroxysteroid dehydrogenase -1 (*HSD11B1*), DNA methyltransferase-1 (*DNMT1*), and DNA methyltransferase-3a (*DNMT3a*) were conducted with the 2ddCT method [44] using cyclophilin-A and beta-actin as reference genes. Relative expression was normalized to control (non-stress) cortex samples.

**Table 1:** Rat RT-PCR primers used in gene expression analyses.

Gene	Gene ID	Primers
Hydroxysteroid dehydrogenase (*HSD11B2*)	NM_017081.1	forward: TGGCAGCCCAGCAGGAGACAT reverse: GCAGCAGTTGCTTGCGCTTCT
Hydroxysteroid dehydrogenase (*HSD11B1*)	NM_017080.2	forward: GGGGCCAGCAAAGGGATCGG reverse: AGGCAGCGAGACACCACCTTCT
DNA methyltransferase (*DNMT1*)	NM_053354.3	forward: GTGGGATGGCTTCTTCAGTA reverse: GGCTTGGTCACAAAACAAAC
DNA methyltransferase (*DNMT3a*)	NM_001003958.1	forward: GGGGCCCCAGCTGAAGGAGA reverse: GCCCCGGGAGCCCTCCATTT
Beta-actin	NM_031144	forward: ATGGATGACGATATCGCTGCG reverse: GGTGACAATGCCGTGTTCAAT
Cyclophillin-A	NM_017101	forward: ATGGTCAACCCCACCGTGTTCTTC reverse: ATCCTTTCTCCCCAGTGCTCAGAG

### DNA Methylation Analysis

**Figure 1:**
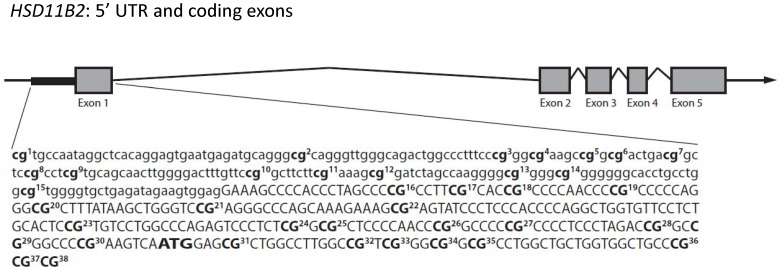
Gene schematic of *HSD11B2* and CpG sites analyzed. The rat *HSD11B2* gene contains a promoter and five exons. The promoter (lowercase letters) and first part of exon 1 (capital letters) are indicated and the ATG site is bolded. 38 CpG sites from −378 to +56 were analyzed for methylation levels and are indicated in bold and numbered.

Tissue (hypothalamus, cortex, placenta) samples from 1 pup per litter from control and stress dams were run in duplicate and included in the DNA methylation analyses. Purified DNA was analyzed (EpigenDX) for CpG methylation by bisulfite pyrosequencing. Samples were bisulfite converted and PCR amplified (see **Table 2** for primers). PyroMark reagents (Qiagen) were used to prepare samples for pyrosequencing (PSQ™96HS). 38 CpG sites in the *Rattus norvegicus HSD11B2* gene (see **Figure 1**) were assayed (−378 to +56, NC_005118.2, ENSRNOT00000023130). The region was assayed by two sets of pyrosequencing primers (see **Table 2**). Paired samples t-test indicated no significant differences between duplicates and average level of methylation across duplicates was used for all subsequent analyses.

**Table 2:** *HSD11B2* Bisulfite Pyrosequencing Primers.

Assay	Primers
PCR Primers (assay 1)	forward: AGGTTTATAGGAGTGAATGAGATG reverse: AACTTTCCTCCACTTCTATCTCAA
PCR Primers (assay 2)	forward: GTTGAGATAGAAGTGGAGGAA reverse: CAAATCTAAACCCAACAACTACA
Sequencing Primers (assay 1)	5′TATAGGAGTGAATGAGATGT3’ 5′GTGTAGTAATTTGGGGA3’
Sequencing Primers (assay 2)	5′TGAGATAGAAGTGGAGGAAA3’ 5′GAGGGTTTAGTAAAGAAA3’ 5′ATTTAGTGTTTTGGTCTAGAG3’

### Sex Determination

In order to determine the sex of pups, DNA purified from placenta of each pup was amplified using primers for *SRY* (AY157669.1; forward: GGAGAGAGGCGCAAGTT GGCT, reverse: GCTATGGTGCAGGGTCGGTCA). 50 ng of DNA was amplified using a PCR amplification kit (Qiagen) according to standardized protocol (15 min at 95C followed by 35 cycles of 94C for 30 s, 60C for 30 s, 72C for 30 s, followed by a single hold at 72C for 10 min) with Sybr DNA loading dye. Immediately after amplification samples were run on a 2% gel and imaged under UV light. Lanes expressing a bright band were identified as male. Multiple *SRY* loci have been identified in the rat genome [45] and some variability in amplified nucleotide length was observed around the expected fragment length of 518 bp.

### Statistics

All statistics were performed using SPSS (IBM, Version 19). Analysis of average ddCT levels to determine differences in gene expression was conducted with a 2-way ANOVA with tissue type and stress treatment as factors. Tukey’s HSD post-hoc tests were used to compare tissue-specific gene expression levels and two-tailed Student’s t-tests were used to determine significant differences associated with maternal stress. Correlation between DNA methylation levels at specific CpG sites was conducted with two-tailed Pearson correlation coefficients. One-way ANOVA was used to compare DNA methylation between tissue types. Repeated measures ANOVA was used to determine the impact of maternal stress on DNA methylation and Tukey’s HSD was used to compare stress effects at specific CpG sites. Analysis of stress effects on overall levels of DNA methylation averaged across sites was conducted using two-tailed Student’s t-tests. Though sex-specific effects on gene expression and DNA methylation were not observed in this sample, sex was used as a covariate in the analyses. Significance was set at the p<0.05 level.

## Results

### Gene Expression in Fetal Hypothalamus, Cortex, and Placenta

**Figure 2:**
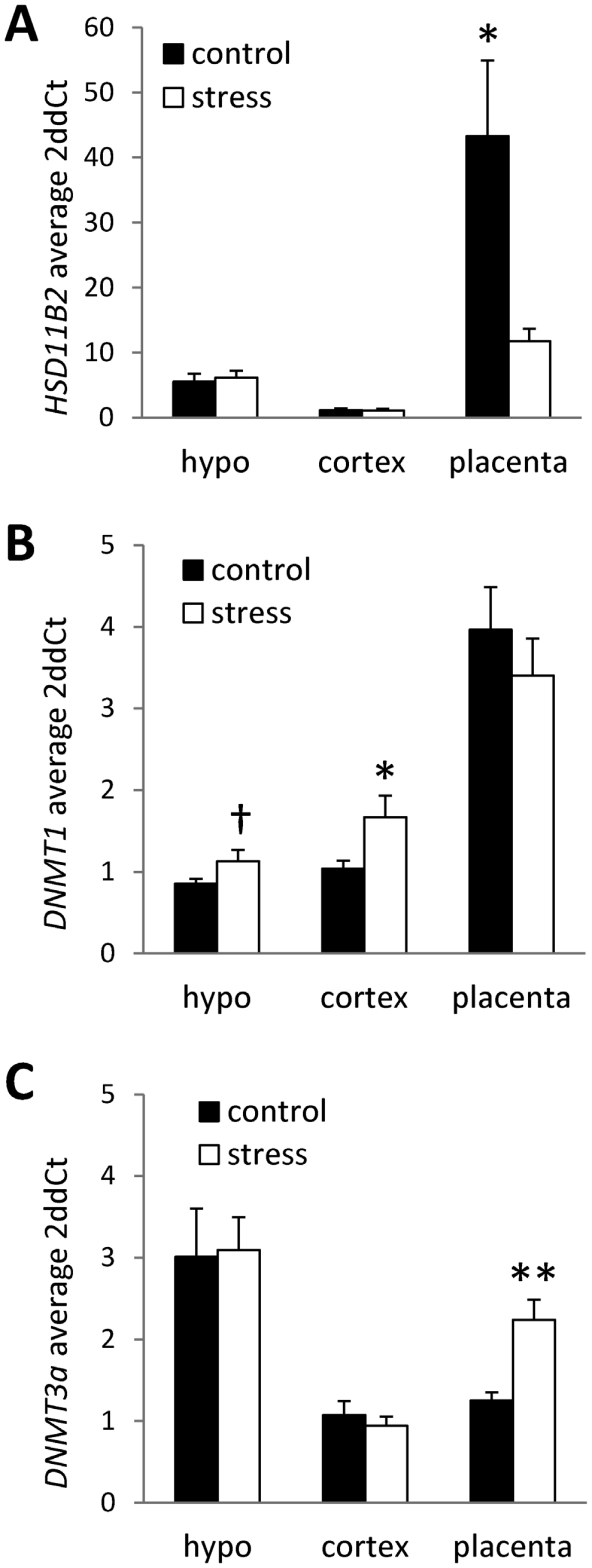
Tissue-specific expression of *HSD11B2* and *DNMTs* in offspring exposed to prenatal stress. Average (mean ± SEM) mRNA levels of (A) *HSD11B2*, (B) *DNMT1*, and (C) *DNMT3a* in hypothalamus (HYPO), cortex, and placenta in control offspring and offspring exposed to prenatal stress. Relative gene expression levels were determined by the 2ddCT method using cyclophilin-A and beta-actin as internal standards. Relative expression was normalized to control (non-stress) cortex samples. (n = 8/group; † p<0.1; *p<0.05, **p<0.01).

Analysis of *HSD11B2* gene expression indicated a main effect of tissue (F(2,40) = 15.37, p<.001), a main effect of stress (F(1,40) = 5.46, p<.05), and a significant tissue by stress interaction (F(2,40) = 6.66, p<.01). Overall, there were significantly higher levels of *HSD11B2* mRNA in the placenta (p<.01) compared to fetal hypothalamus and cortex (placenta: M = 28.57±7.38; hypothalamus: M = 5.85±.77; cortex: M = 1.13±.17). Prenatal stress was associated with a significant decrease in *HSD11B2* mRNA in the placenta (compared to controls, p<.05; **Figure 2a**) and no group differences in *HSD11B2* mRNA were evident in the fetal hypothalamus or cortex. The stress-induced down-regulation of placental 11β-HSD was specific to *HSD11B2* as analysis of *HSD11B1* mRNA levels indicated no significant effects of stress and no stress by tissue type interactions on the expression of this gene. Based on the tissue-specific effects of stress on *HSD11B2* gene expression, we proceeded to determine whether there were tissue specific effects of stress on the expression of genes involved in epigenetic regulation of gene expression (*DNMT1* and *DNMT3a*). Overall, levels of *DNMT1* mRNA were found to be significantly higher in the placenta compared the fetal hypothalamus and cortex (p<.001) and levels of *DNMT3a* mRNA were significantly elevated in the hypothalamus compared to the cortex and placenta (p<.001; see **Figure 2**). Within the hypothalamus, we found a trend for increases in *DNMT1* gene expression amongst prenatally stressed offspring (t(14) = −1.79, p = .09). Within the fetal cortex, *DNMT1* expression was significantly increased in stressed offspring (t(14) = −2.24, p<.05; **Figure 2b**). There were no significant effects of stress on *DNMT1* expression in the placenta. Analysis of the *de novo* methyltransferase *DNMT3a*, indicated a placenta-specific effect of stress on the expression of this enzyme. Prenatal stress was found to be associated with increased placental *DNMT3a* mRNA levels (t(14) = −3.71, p<.01; **Figure 2c**).

### Comparison of CpG Methylation of the HSD11B2 Gene in Fetal Hypothalamus, Cortex, and Placenta

The *HSD11B2* promoter and region from −378 to +56 is rich in CpG sites and contains several transcription factor binding sites for Sp1 and NF-κB, suggesting that this region would be a likely candidate for regulation of gene transcription by DNA methylation. Moreover, previous studies in rats report environmentally-induced differences in methylation within this region associated with variation in gene transcription [41]. Analysis of within-individual variation in CpG methylation of the *HSD11B2* promoter (and at sites within the first exon – see **Figure 1**) generally indicated a lack of correlation in the methylation status of sites between different tissues. However, at sites 12 (r(8) = .81, p<.01), 34 (r(8) = .81, p<.01), and 36 (r(8) = .72, p<.05), placental CpG methylation levels were strongly positively associated with fetal cortex CpG methylation levels, but neither placenta nor fetal cortex CpG methylation levels correlated with hypothalamic CpG methylation levels at these sites. Comparison of average CpG methylation levels at specific sites within the *HSD11B2* promoter across tissue types indicated several sites that where differentially methylated between the hypothalamus, cortex, and/or placenta (**Table 3**). At sites 1 and 3 (site 1: F(2,23) = 114.56, p<.001; site 3: F(2,23) = 8.20, p<.01), there was elevated methylation in placenta compared to both hypothalamus and cortex (p<.01). At site 5 (F(2,23) = 7.61, p<.01), there was elevated methylation in placenta and hypothalamus compared to cortex (p<.05). At sites 10 and 14 (site 10: F(2,23) = 4.15, p<.05; site 14: F(2,23) = 8.01, p<.01), there were elevated levels of methylation in hypothalamus compared to cortex (p<.05). At all other CpG sites, there were no differences between tissue types in the average level of DNA methylation.

**Table 3:** Tissue-specific differences in % CpG methylation within the *HSD11B2* gene promoter.

CpG Site	Hypothalamus	Cortex	Placenta
1	2.21±.24	2.34±.78	14.82±.84**
3	2.58±.46	2.46±.30	4.27±.29**
5	2.78±.59	0.96±.33*	3.78±.59
10	3.54±.13*	2.28±.33	2.93±.40
14	5.57±.28*	3.84±.39	4.56±.23

* p<.05, **p<.01.

### Impact of Prenatal Stress on HSD11B2 CpG Methylation

**Figure 3:**
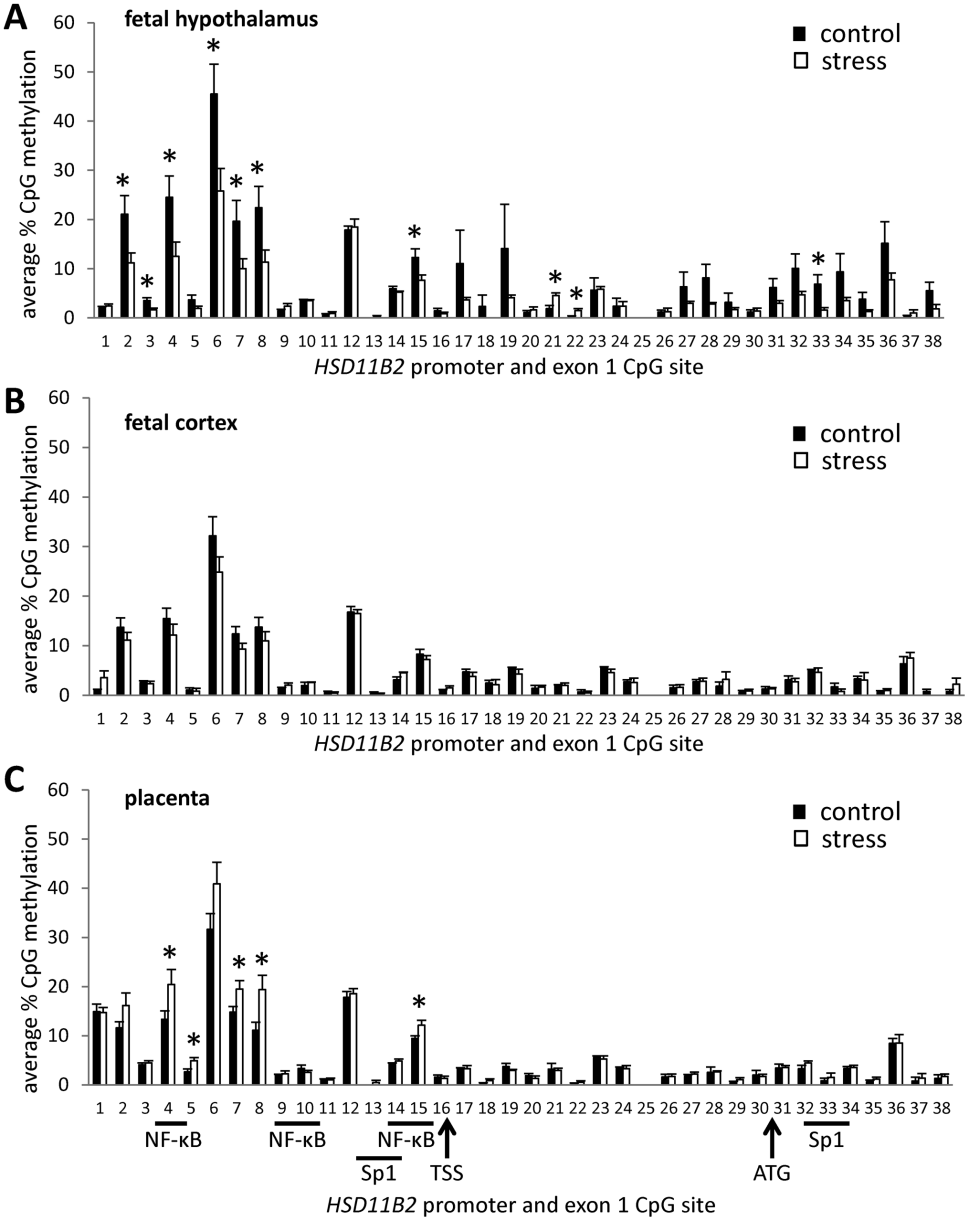
Tissue-specific effects of prenatal stress on DNA methylation of the *HSD11B2* promoter and exon 1. Average (mean ± SEM) % DNA methylation at 38 CpG sites in the *HSD11B2* promoter and exon 1 in (A) fetal hypothalamus, (B) fetal cortex, and (C) placenta of control offspring and offspring exposed to prenatal stress. Location of Sp1 and NF-κB transcription factor binding sites indicated, as well as the transcription start site (TSS) and ATG start codon. (n = 4/group run in duplicate; *p<0.05).

Analysis indicated main effects of tissue [F(2,17) = 4.67, p<.05], stress [F(1,17) = 4.54, p<.05], and a significant tissue by stress interaction [F(2,17) = 7.76, p<.01] on CpG methylation within the *HSD11B2* promoter. Within the fetal hypothalamus (**Figure 3a**), prenatal maternal stress was associated with reduced CpG methylation at sites 2, 3, 4, 6, 7, 8, 15, and 33 and elevated CpG methylation at sites 21 and 22. Within the fetal cortex (**Figure 3b**), there was no effect of prenatal maternal stress on CpG methylation. In the placenta (**Figure 3c**), prenatal maternal stress was associated with increased CpG methylation at sites 4, 5, 7, 8, and 15. Stress-induced changes in hypothalamic and placental CpG methylation of the *HSD11B2* promoter were most evident when examining CpG site-specific DNA methylation, though analysis of the effects of stress on average methylation across the 15 CpG sites within the promoter region indicated a trend for decreased methylation in the hypothalamus (M±SEM; control: 12.26%±1.75, stress: 7.66%±1.05; p = .06) and increased methylation within the placenta (control: 9.47%±.53, stress: 12.15%±1.00; p = .06).

## Discussion

In the current study we report a robust and tissue-specific effect of maternal stress during pregnancy in rats on mRNA levels of the enzyme *HSD11B2*. Stress, anxiety, and depression during pregnancy can have a long-lasting impact on the psychological health of children [4], [46]. Chronic unpredictable prenatal stress in rodents has been shown to impact cognitive and stress-coping behaviors, particularly when stress is experienced in the third gestational week [47]. The confinement stress employed in the current study was mild but daily and unpredictable over the third gestational week in order to mimic the characteristics of chronic mild stress/anxiety experienced by human mothers. Consistent with previous findings, chronic stress was associated with decreased transcription of *HSD11B2* within the placenta in late gestation [12], [26]. Maternal stress was also found to induce increases in the transcription of the DNA methyltransferases *DNMT1* and *DNMT3a*. Within the placenta, this stress effect was specific to the *de novo* methyltransferase *DNMT3a*, whereas in fetal hypothalamus and cortex, prenatal stress induced increased mRNA levels of *DNMT1*. Consistent with the reduced *HSD11B2* mRNA in placenta, we find increased placental CpG methylation within several sites of the *HSD11B2* gene promoter associated with maternal stress. In contrast, our analysis indicates decreased DNA methylation at several sites of the *HSD11B2* gene promoter in fetal hypothalamic tissue associated with stress and no effect of this prenatal exposure on DNA methylation in the fetal cortex. These findings highlight the tissue specificity of epigenetic effects. However, we do identify several CpG sites within the placenta at which an individual’s DNA methylation levels do significantly predict those observed in the fetal cortex, raising the intriguing possibility of using the epigenetic status of placenta to predict corresponding changes in the brain. Overall, these findings provide novel evidence for the epigenetic regulation of *HSD11B2* as a potential mechanism linking maternal stress during gestation, dysregulation of placental gene expression, and neurodevelopmental outcomes in offspring.

Though placental 11ß-HSD2 can function as an enzymatic buffer against the deleterious effects of exposure to maternal glucocorticoids, it is clear that this enzyme can be down-regulated by adverse prenatal experiences [26], [28], [29], [48], thus limiting its capacity to protect the developing fetus. However, the nature and timing of these experiences is an important consideration in predicting the direction of the effects on 11ß-HSD2. For example, acute stress in pregnant rats has been found to increase the activity of placental 11ß-HSD2 [48] and similarly in humans, betamethasone treatment within 72 hours of parturition leads to increased 11ß-HSD2 enzymatic activity [49]. Up-regulation of placental 11ß-HSD2 may thus be an adaptive response to elevated maternal glucocorticoids. In contrast, chronic exposure to maternal glucocorticoids may lead to reduced placental 11ß-HSD2, increased exposure of fetal tissues to glucocorticoids, and neurodevelopmental and metabolic phenotypes associated with glucocorticoid programming. Heightened glucocorticoid exposure during fetal development may promote lung development and thus increase survival following pre-term birth – an obstetric outcome predicted by maternal stress [50], [51]. Thus, though heightened anxiety and HPA activity may be considered maladaptive, these outcomes may be the cost of promoting survival amongst the offspring of gestationally stressed females. One possible strategy to buffer the brain against these neurodevelopmental consequences would be to increase 11ß-HSD2 within the brain. Though we do not find an increase in hypothalamic or cortical *HSD11B2* mRNA levels, the stress-induced DNA hypomethylation of this gene that we observe in hypothalamic tissue may be an epigenetic precursor to these buffering effects. Time course analysis of *HSD11B2* gene expression and DNA methylation throughout the prenatal stress exposure and into the postnatal period may thus be a key strategy for determining the dynamics of glucocorticoid programming mechanisms.

Investigation of the epigenetic regulation of *HSD11B2* has previously been explored outside the context of studies on maternal stress. In humans and in rats, the promoter and first exon of the *HSD11B2* gene (regions analyzed in the current study) are rich in CpG sites and DNA methylation levels at CpG sites within this region are related to the expression of this gene [52]. Pharmacological hypomethylation of *HSD11B2* through *in vitro* or *in vivo* treatment with the DNA methyltransferase inhibitor 5-aza-2′-deoxycytidine has been found to increase *HSD11B2* expression [52]. One functional consequence of elevated DNA methylation is the inhibition of transcription factor binding to the promoter regions of target genes and in the case of *HSD11B2*, CpG methylation at *Sp1* and *NF1* recognition sequences prevents binding of these transcription factors and diminishes the transcriptional activity of *HSD11B2* [52]. Increased hepatic *HSD11B2* promoter methylation and decreased *HSD11B2* mRNA has also been observed in neonatal offspring exposed to *in utero* magnesium deficiency [53]. In rats, intrauterine growth restriction is associated with both reduced *HSD11B2* expression and increased *HSD11B2* promoter methylation in kidneys at birth and these epigenetic effects lead to altered transcription factor binding of Sp1 and NF-κB [41]. These epigenetic effects were found to be sex-specific and present both at birth and postnatal day 21, indicating long-term consequences of prenatal *HSD11B2* dysregulation. The sex-specificity of environmentally induced changes in DNA methylation is increasingly evident and indeed epigenetic modifications may be involved in the normal process of sexual differentiation [9], [54], [55]. Though we do not find sex-specific effects of maternal stress on *HSD11B2* expression or DNA methylation in the current study, it may be that large samples are needed to detect sex-differences in the epigenetic regulation of this gene (to account for individual differences in hormonal exposure) or that sex-differences are more likely to emerge in later development.

A candidate mechanism for the increased placental DNA methylation and increased *HSD11B2* levels we have found in response to maternal stress during pregnancy may involve the up-regulation of DNA methyltransferase levels in the placenta of stressed offspring. Our data indicate a placenta-specific increase in *DNMT3a* mRNA in response to maternal stress. This stress-induced effect has implications for genome-wide epigenetic changes, and may account for the diverse phenotypic outcomes associated with maternal adversity during pregnancy. DNMT1 and DNMT3a are enzymes active throughout the lifespan and thus could potentially serve as a mechanism for long-term epigenetic regulation, though it is possible that DNMT3b (only active early in development) is similarly altered by prenatal stress to mediate *HSD11B2* promoter methylation. Our data are consistent with previous findings indicating an up-regulation of DNMTs in the placenta of mice exposed to 1^st^ trimester maternal stress [9]. Targeted deletion of *DNMT1* or *DNMT3a* in mice has been found to produce embryonic and postnatal lethality and widespread epigenetic changes – particularly amongst imprinted genes – in embryonic tissues [56]–[58]. Amongst offspring that are heterozygous for a mutation in DNA methyltransferase 3-like (DNMT3L) protein, there are specific disruptions to placental development [59]. DNMT3L interacts directly with DNMT3a to facilitate DNA methylation [60], and within the placenta, epigenetic regulation of *DNMTs* and *DNMT3L* may account for the changing epigenetic and transcriptional profiles in this tissue during the course of pregnancy [61].

Within the fetal hypothalamus and cortex we observe discordance in the relationship between *HSD11B2* mRNA, DNMT expression, and CpG methylation within the *HSD11B2* gene promoter. Despite stress induced decreases in *HSD11B2* CpG methylation in the fetal hypothalamus, we do not find differential expression of hypothalamic *HSD11B2* and within the fetal cortex we find stress-induced increases in *DNMT1* without corresponding changes in the DNA methylation or expression of *HSD11B2*. The regulation of *HSD11B2* expression is complex and these paradoxical findings suggest that gene regulation in response to prenatal stress is also accomplished by other epigenetic mechanisms, such as chromatin remodeling, as well as regulation by transcription factors known to influence *HSD11B2* transcription (such as Sp1 and NF-κB, see **Figure 3**). These results illustrate the challenge of *in vivo* studies of epigenetic effects and the complexity of the pathways through which transcriptional activation is achieved. Determining the temporal dynamics of stress-induced changes in DNMTs, DNA methylation, and gene expression in different tissue types may provide some insight into this complexity. For example, elevations in DNMT3a and DNMT1 will likely precede elevated methylation of target genes, and the reduced fetal CpG methylation observed in hypothalamic tissue of stressed offspring will likely precede observable changes in mRNA in this brain region. The association of DNMT3a with *de novo* methylation and of DNMT1 with maintaining methylation marks in dividing cells adds a further layer of temporal complexity. Though *in vivo* imaging of these changes within individuals is not methodologically feasible, a cross-sectional design including offspring of varying embryonic and postnatal ages would help elucidate these dynamics. In addition, it is important to consider the diversity of epigenetic mechanisms that can contribute to maternal stress effects on gene regulation, particularly histone modifications, and to further explore other physiological factors that have been demonstrated to alter *1HSD11B2* activity and expression, such as catecholamines [62] and proinflammatory cytokines [63].

The translation of *in vitro* and animal studies of environmentally-induced epigenetic variation in target genes may provide critical insights into the role of these mechanisms in long-term disease risk. In humans, maternal antenatal depression has been found to predict elevations in DNA methylation of the glucocorticoid receptor gene (*NR3C1*) promoter in fetal cord blood samples and the degree of CpG methylation detected in these cells predicts salivary cortisol levels of infants at 3 three months of age [64]. Maternal stress in the form of intimate partner violence has similarly been found to predict *NR3C1* DNA methylation in blood samples from children/adolescents that were *in utero* during the stressor [65]. In both cases, these studies have used peripheral tissues as a proxy for potential epigenetic changes that may be induced within the brain and account for elevations in the HPA response to stress associated with these adverse prenatal exposures. However, it is important to note that the relationship between epigenetic variation in peripheral/available tissues (*e.g*. blood, placenta) and target tissues (*e.g.* brain, organs) has yet to be established and will likely vary dependent on the target gene, the nature of the environmental exposure, and the timing of sampling. Our data indicate that though at most CpG sites within the *HSD11B2* gene promoter, DNA methylation levels are equivalent across placenta, hypothalamus, and cortex, within-individual correspondence in the methylation patterns between these tissues is limited. Though it may be possible to predict brain DNA methylation from the degree of CpG methylation observed in placenta – the biological relevance of the epigenetic markers identified in the current study has yet to be established. Future studies are needed both in humans and animals to address the issue of tissue specificity in environmentally-induced epigenetic variation and to elucidate the mechanisms contributing to the concordance and discordance in tissue-specific epigenetic profiles.
